# Optimizing the Interface between Hole Transporting Material and Nanocomposite for Highly Efficient Perovskite Solar Cells

**DOI:** 10.3390/nano9111627

**Published:** 2019-11-16

**Authors:** Zeinab Safari, Mahmood Borhani Zarandi, Antonella Giuri, Francesco Bisconti, Sonia Carallo, Andrea Listorti, Carola Esposito Corcione, Mohamad Reza Nateghi, Aurora Rizzo, Silvia Colella

**Affiliations:** 1 Department of Physics, Yazd University, P.O. Box 89195-741, Yazd 89195-741, Iran; zsafari@yahoo.com (Z.S.); mborhani@yazd.ac.ir (M.B.Z.); 2 Dipartimento di Ingegneria dell’Innovazione, Università del Salento, via per Monteroni, km 1, 73100 Lecce, Italy; antonella.giuri@unisalento.it; 3 Istituto di Nanotecnologia CNR-Nanotec, Polo di Nanotecnologia c/o Campus Ecotekne, via Monteroni, 73100 Lecce, Italy; francesco.bisconti.90@gmail.com (F.B.); sonia.carallo@nanotec.cnr.it (S.C.); andrea.listorti@unisalento.it (A.L.); aurora.rizzo@nanotec.cnr.it (A.R.); silvia.colella@unisalento.it (S.C.); 4 Department of Chemistry, Yazd Branch, Islamic Azad University, Yazd 8915 813135, Iran; m_nateghi60@hotmail.com

**Keywords:** organometallic halide perovskite, polymeric hole transporters, starch composite, solar cells, interfaces

## Abstract

The performances of organometallic halide perovskite-based solar cells severely depend on the device architecture and the interface between each layer included in the device stack. In particular, the interface between the charge transporting layer and the perovskite film is crucial, since it represents both the substrate where the perovskite polycrystalline film grows, thus directly influencing the active layer morphology, and an important site for electrical charge extraction and/or recombination. Here, we focus on engineering the interface between a perovskite-polymer nanocomposite, recently developed by our group, and different commonly employed polymeric hole transporters, namely PEDOT: PSS [poly(3,4-ethylenedioxythiophene):poly(styrenesulfonate)], PEDOT, PTAA [poly(bis 4-phenyl}{2,4,6-trimethylphenyl}amine)], Poly-TPD [Poly(*N*,*N*′-bis(4-butylphenyl)-*N*,*N*′-bis(phenyl)-benzidine] Poly-TPD, in inverted planar perovskite solar cell architecture. The results show that when Poly-TPD is used as the hole transfer material, perovskite film morphology improved, suggesting an improvement in the interface between Poly-TPD and perovskite active layer. We additionally investigate the effect of the Molecular Weight (MW) of Poly-TPD on the performance of perovskite solar cells. By increasing the MW, the photovoltaic performances of the cells are enhanced, reaching power conversion efficiency as high as 16.3%.

## 1. Introduction

Organometal halide perovskite solar cells (PSCs) are regarded as the most promising third-generation photovoltaic technology [1,2,3,4,5,6], thanks to their stunning characteristics i.e., very high absorption coefficient; small exciton binding energy; long charge carrier diffusion length as well as solution processability [7,8,9,10]. Up to now, power conversion efficiency (PCE) has significantly increased, from 3.8% since then to currently 25.2% [11,12]. Engineering the architecture of solar cell devices to improve performance, stability, and to reduce the cost of manufacture, are the challenging issues in the field. So far, different structures of PSCs have been reported such as mesoporous and planar layouts [13,14,15]. The planar PSCs are further divided into direct (n-i-p) or inverted (p-i-n) structures based on the deposition sequence of charge transport materials and perovskite [16]. Inverted structures typically use organic transport layers, and possess some merits such as low-temperature processing, easy-fabrication, high throughput and cost-effectiveness for industrial application [13,16,17].

The next technological challenge is finding a scalable solar cell production method possibly at low cost and mild temperature of processing. In this scenario, recent advances in the development of inverted device architecture with polymeric interlayers [18,19] and the use of molecular additives to simplify the solution processing conditions [20,21] and to improve the resulting perovskite material properties [20] provide a further major stimulus to the research in field.

Among the various deposition methods indeed, vapor-assisted solution processing (VASP) [22], physical vapor deposition (PVD) [23], single step route [24] and two-step [25,26] or sequential deposition route through the solution of depositing metal halide perovskite films, the solution processing is simpler, cheaper, and more suitable for large scale production [27,28]. However, in this method, there are many structural defects along the grain boundaries of polycrystalline perovskite films [29,30,31,32]. So far, the inclusion of various additives to control perovskite crystalline structure, morphology, and moisture stability has been successfully investigated [33,34,35,36,37,38,39]. One of the most attractive approaches is the use of polymeric templating agents to grow perovskite films, for various reasons. First of all, it has been demonstrated to improve the stability of the perovskite material and to make its processing more facile and compatible with large-scale manufacturing [40,41,42].

Very recently, the addition of starch in the perovskite precursor solution allowed to conveniently tune the solution viscosity, enabling the deposition of a thick and continuous perovskite film with a simple single spin-coating step, with obvious positive implications on the processing of the whole device. In fact, the presence of starch in the precursor of perovskite solutions improved crystal size and film morphology and allowed to eliminate the solvent dripping step, which is hardly scalable on large area production and generally employs toxic solvents. In this approach, instead, only Dimethyl sulfoxide DMSO is used as solvent [41,43,44].

If on one hand the use of DMSO and of the bio-polymer starch reduces the environmental impact of the process, on the other hand this might change the interaction with the layers underneath in the device architecture, with obvious implications on the wettability, the charge transporting layer/active material interface and, overall, on the device performance. In fact, it is well known how the charge transport across the interfaces, hysteresis phenomena [45], but also environmental stability [46] dramatically depend on this interface [47], the reason why it is of paramount importance to optimize the interface where the perovskite grows, especially in the case of new solvents/perovskite solutions employed [43,48,49,50].

The chosen charge transporting layers play a significant role in the field of interface engineering, [51] and a large number of materials are currently available for implementation in the device structure [52,53]. A typical inverted architecture includes a polymeric transporting layer (HTL) underneath the perovskite film, and a fullerene derivative, such as [6,6]-phenyl C_61_ butyric acid methyl ester PCBM, as the electron transporting layer [54]. The polymeric hole transporting material HTM, in particular, plays a preeminent role in influencing the growth of the perovskite layer on top of the corresponding interface. PEDOT: PSS has first been used as HTM. [55]. However, the maximum Voc that could be reached for PSCs was 0.8 V due to the energy levels and the partial solubility in the perovskite polar solvents (DMSO, Dimethylformamide (DMF)) that does not allow the obtainment of a sharp interface [41,56]. Among the most used and efficient polymers, PTAA presented a record of PCE = 22.1% on a laboratory scale of <0.1 cm^2^ device area [57] with Voc = 1.14 V. In fact, the amorphous character of such molecular structure leads to uniform and smooth thin films and therefore one can expect high-value isotropic carrier transport [57,58,59]. [Poly(*N*,*N*′-bis(4-butylphenyl)-*N*,*N*′-bis(phenyl)-benzidine] (Poly-TPD) has also been extensively applied as HTMs thanks to the favorable lowest unoccupied molecular orbital (LUMO). Although, at first Poly-TPD has been used as interlayer at the interface between the conventional PEDOT: PSS HTL and the CH_3_NH_3_PbI_3_ perovskite to obtain better matching in the perovskite valence band [60].

Here, we selected different HTM materials, namely the already mentioned PEDOT: PSS, PEDOT, PTAA, and Poly-TPD, to study interface engineering between HTM and the nanocomposite based on methyl ammonium lead iodide perovskite (MAPI): Starch in an inverted fully organic architecture Indium tin oxide ITO/HTM/MAPI: Starch/PCBM/Bathocuproine (BCP)/Al, with the aim of optimizing the performances of the PSC. We found that PEDOT: PSS is not compatible due to the damage caused by the pure DMSO solvent, making the use of hydrophobic HTM necessary. Among the ones employed in this work, poly-TPD was found to be the best choice, likely due to its favorable energy levels allowing for a higher Voc. In order to further optimize the efficiency, we evaluated the effect of different post-deposition treatments, UV-Ozone UVO, and oxygen plasma, on the highly hydrophobic poly-TPD layer, to improve its wettability to the perovskite precursors, as well as the effect of the different molecular weights, ranging from MW < 10 kDa to MW > 20 kDa. We found that a higher MW slightly improves the PV performances, while the oxygen plasma appeared to damage the polymer surface even at very low powers, allowing UVO to be selected as the best post deposition treatment.

## 2. Experimental Section

### 2.1. Materials

Lead (II) iodide (PbI_2_, ultradry 99.999% metals basis) and methylammonium iodide CH3NH3I (MAI) was purchased from Alfa Aesar (Kandel, Germany) and GreatCell Solar (Rome, Italy), respectively. Dimethyl sulfoxide anhydrous, 99.9% (DMSO), chlorobenzene anhydrous, 99.8% (CB), 2-isopropanol (IPA), lithium fluoride 99.99%, PTAA [poly(bis{4-phenyl}{2,4,6-trimethylphenyl}amine)] and bathocuproine 96% (BCP) were purchased from Aldrich (Milan, Italy). Corn starch (Maizena) was supplied from Unilever (Rome, Italy). PEDOT: PSS (Clevios PVP AI4083) and PEDOT (Clevios HTL Solar 3) were supplied from Heraeus (Hanau, Germany). Poly[*N*,*N*′-bis(4-butylphenyl)-*N*,*N*′-bis(phenyl)benzidine](poly-TPD), was supplied from Lumtec (New Taipei city, Taiwan). [6,6]-phenyl C_61_ butyric acid methyl ester (PCBM) was purchased from Nano-c. All the materials were used as received without any further purification.

### 2.2. MAPI: Starch Based Solution Preparation

The perovskite used was prepared by following the method developed in our previous work [43]. In detail, equimolar stoichiometry perovskite precursors (MAI:PbI_2_ = 1:1) were solubilized in DMSO, at 80 °C for 30 min, with precursor concentration of 30 wt%. Subsequently, 15 wt% of starch was added to perovskite precursors followed by stirring at 80 °C for 5 h. All the solutions were prepared in a N_2_ filled glovebox.

### 2.3. Oxygen Plasma Treatment

Zepto oxygen plasma cleaner was used to treat the Poly-TPD film. The radio frequency (RF) level was turned to minimum and power was kept at 40 W. All treatments were performed for 5 s.

### 2.4. Characterization

The contact Angle Measurement: a First Ten Angstroms FTA1000 Quick Start (Milan, Itay) instrument was used to analyze the wettability of the different HTM substrates.

Atomic Force Microscopy (AFM): a Park Scanning Probe Microscope (PSIA) (Santa Clara, CA, USA) operating in air at room temperature and in a noncontact mode (in order to reduce tip induced surface degradation) was used.

Scanning Electron Microscopy (SEM): the morphology of the films was analyzed by using Carl Zeiss Auriga40 Crossbeam (Oberkochen, Germany) instrument in high vacuum and high-resolution acquisition mode. SEM was equipped with Gemini column and an integrated high efficiency in-lens detector. The process was performed at 5 kV as the desired acceleration voltage.

### 2.5. Device Fabrication and Characterization

We sequentially cleaned the 15 × 15 mm^2^ glass ITO patterned substrates (Visiontek Systems Ltd., Chester, UK) by ultrasonication them in acetone and deionized water.

Subsequently, the cleaned ITO glasses were treated with O_2_ plasma for 3 min. The different hole transport materials were deposited in air as described in the following.

About 40 μL of PTAA (1.5 mg/mL in toluene) solution was spin coated at 6000 rpm for 30 s and annealed at 100 °C for 10 min in order to obtain a 35 nm thick. Then, 100 μL of PEDOT: PSS and PEDOT commercial solutions were spin coated after filtration with proper filter at 3000 and 8000 rpm, respectively, for 60 s and annealed at 140 °C for 15 min, achieving corresponding thicknesses of 35 and 45 nm.

A total of 40 μL of Poly-TPD (1.5 mg/mL in chlorobenzene) solution was spin coated at 4000 rpm for 60 s giving a thickness of about 35 nm. In order to improve the surface wettability, the poly-TPD film was exposed under UVO for 3 min [43].

The device fabrication process was completed in a glovebox. Based on the work reported in [43], perovskite: Starch nanocomposite film was prepared by spin coating the solution at 9000 rpm for 20 s on top of the hole transport materials and finally annealed at 100 °C for 30 min.

We deposited about 50 nm of PCBM layer by spin coating 100 μL of filtered solution (25 mg/mL in chlorobenzene) on the active layer at 1000 rpm for 60 s following by the deposition of a few nanometers thin layer of BCP by spin coating the solution (0.5 mg/mL in isopropanol) at 6000 rpm for 20 s.

In the final step, 100 nm of Al electrodes were evaporated through shadow mask in high vacuum. The active area was 0.04 cm^2^.

The device characterization was performed using a Keithley 2400 Source Measure Unit and Air Mass 1.5 Global (AM1.5G) solar simulator (Newport 91160A) exposed to irradiation intensity of 100 mW/cm^2^. Current-voltage characteristics of the devices were obtained in the range from 1.2 to 0.2 V.

## 3. Results and Discussion

**Figure 1:**
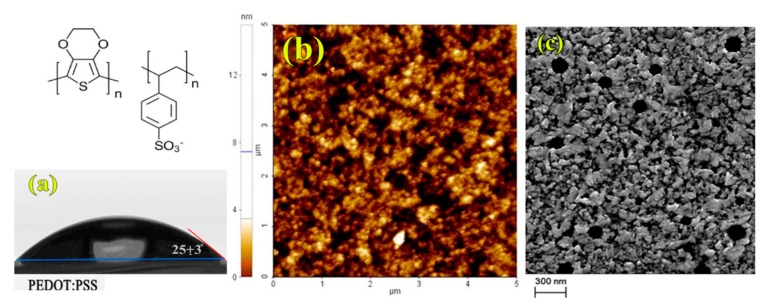
Water contact angle on PEDOT: PSS deposited on Glass/Indium Tin oxide (ITO) (**a**). Atomic Force Microcopy (AFM) images (5 × 5 nm) of Perovskite/starch film deposited on PEDOT: PSS film (Rq = 1.11 nm) (**b**). A Scanning Electron Microscopy (SEM) image of perovskite/starch deposited on PEDOT: PSS film (**c**).

The need to explore different HTM for the specific mixture MAPI: Starch, solubilized in pure DMSO, starts from the evidence of a poor resistance of the PEDOT: PSS layer to this solvent. Clearly, PEDOT: PSS exposes a quite hydrophilic surface, as shown by the contact angle measurement (25° ± 3°) reported in Figure 1a, and due to its solubility in water, it is easily dissolved during the spin-coating of perovskite precursor solutions. In fact, AFM and SEM images of the perovskite film deposited on top of the PEDOT: PSS layer, respectively in Figure 1b,c, show the presence of round shaped aggregates that have been attributed to a damaging of the PEDOT: PSS layer by the DMSO solvent, resulting eventually in a desorption of PSS aggregates [43].

**Figure 2:**
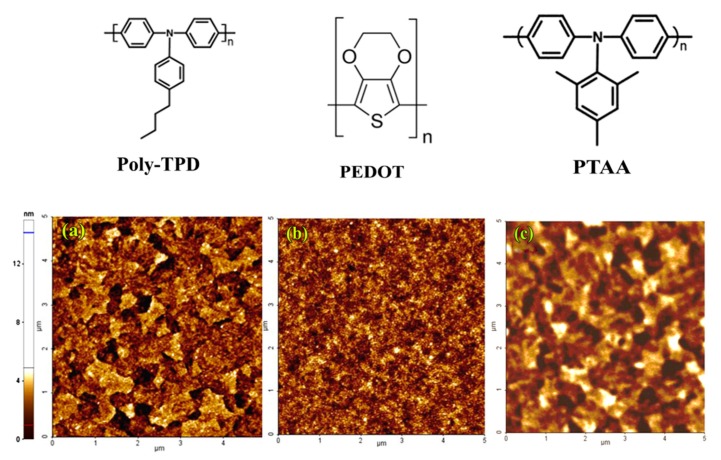
AFM images (5 × 5 μm) of Poly-TPD (Rq = 1.72 nm) (**a**), PEDOT (Rq = 3.10 nm) (**b**), and PTAA (Rq = 1.01 nm) (**c**).

**Figure 3:**
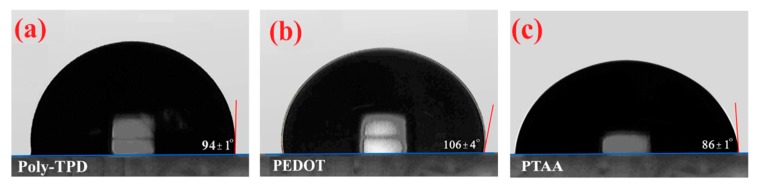
Water contact angle of the p-type polymer films of interest: Poly-TPD (**a**), PEDOT (**b**), and PTAA (**c**).

To solve this issue, we modified solar cell device architecture, replacing PEDOT: PSS with alternative polymeric hole transporting material not soluble in DMSO, namely PEDOT [poly(3,4-ethylenedioxythiophene)], PTAA, and Poly-TPD, whose structures are sketched in Figure 2. Firstly, we characterized these polymer layers deposited on glass/ITO, by analyzing the morphology (AFM images in Figure 2) and their wettability properties (Figure 3).

All the employed polymers show a much higher hydrophobicity with respect to PEDOT: PSS. Their contact angles shown in Figure 3a–c, in fact, are, respectively, 94° ± 1°, 106° ± 3°, and 86° ± 1°.

Despite the high hydrophobicity of the employed polymers, the use of starch aids the increase of the viscosity of the perovskite precursor solution, guaranteeing a homogeneous and reliable deposition of perovskite films onto these untreated alternative layers. Only Poly-TPD requires a mild treatment under ultraviolet-ozone (UVO), as commonly done in the literature [61], to allow the formation of a compact perovskite film.

In the deposition process of such perovskite: Starch nanocomposite [43] on the different selected polymers, in a single spin-coating step, the good wettability of the perovskite precursors solution, thus a good coverage of the substrate, are fundamental to achieve a good film morphology.

**Figure 4:**
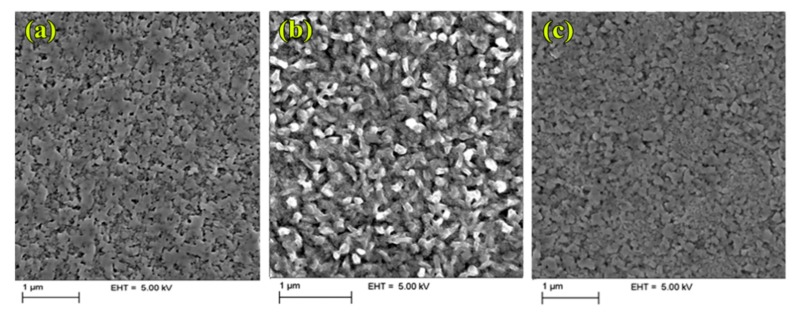
Top view SEM images of perovskite/starch crystals grown on Poly-TPD (**a**), PEDOT (**b**), and PTAA (**c**).

The SEM analysis of the solution processed MAPI: Starch films on Poly-TPD, PEDOT and PTAA are shown in Figure 4. Unlike PEDOT: PSS, a homogeneous perovskite surface, without round shaped aggregates, was observed for all the polymeric substrate investigated, confirming our previous observation. However, a different morphology of the MAPI: Starch film was evidenced when the nanocomposite is deposited on PEDOT (Figure 4c) compared to PTAA and poly-TPD. In detail, small and long pillar-like grains were observed in the first case compared to smooth surface with larger grain size for the MAPI: Starch film deposited on PTAA and Poly-TPD.

### Inverted Perovskite Solar Cells Performance

**Figure 5:**
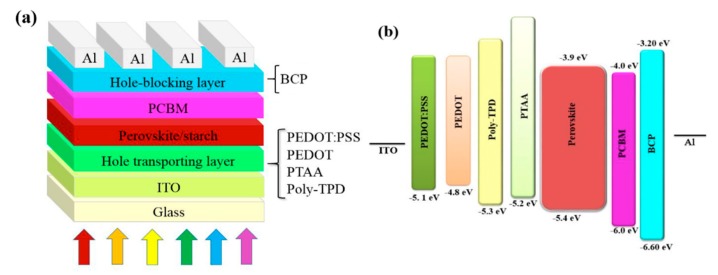
Structure of perovskite solar cells. (**a**) Device architecture and (**b**) energy-band diagram of the devices with PEDOT: PSS, PEDOT, Poly-TPD, and PTAA as the HTLs.

With the aim to investigate the influence of the different HTM/MAPI: Starch interfaces and perovskite: Starch morphologies on the performances of the device, we fabricated inverted perovskite solar cells with the architecture ITO/HTM/MAPI: Starch/PCBM/BCP/Al shown in Figure 5a. The energy-band diagram of perovskite solar cells is shown in Figure 5b.

The average measured photovoltaic parameters (under simulated one sun AM1.5G irradiation) are summarized in Table 1.

Both sets of cells with PEDOT and Poly-TPD had average fill factors (FF) of up to 70% with average FFs above with 71.1% and 72.4%, respectively. These high values indicate a low resistance between the ITO, the HTL, and the MAPI/starch.

**Figure 6:**
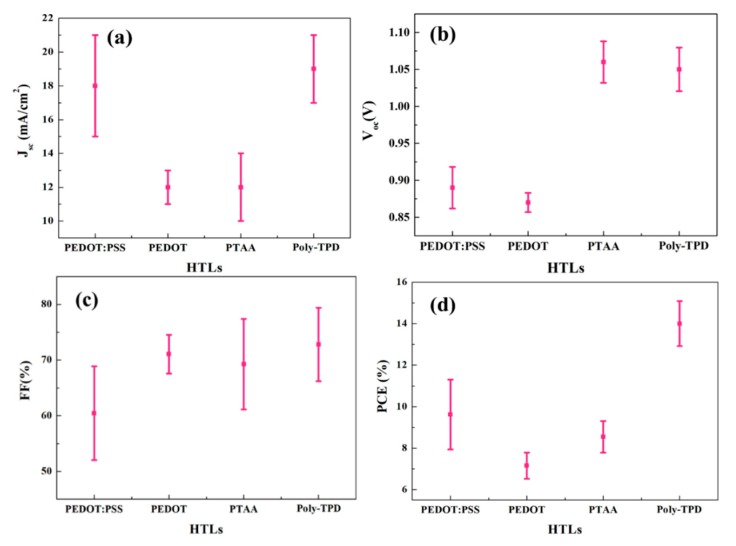
Statistics of photovoltaic parameters, (**a**) fill factors (FF), (**b**) V_oc_, (**c**) J_sc_, and (**d**) power conversion efficiency (PCE) for different HTLs.

Remarkably, the Voc of cells with PTTA and Poly-TPD hole-transporting layers lies within a narrow margin 1.06 and 1.05 V, despite severe differences in the chemical structure and energetics of the used polymers. However, the current in cells with PTAA is much less than cells with Poly-TPD. Several studies in the literature have shown that the competition between free charge generation and recombination in any type of solar cell will challenge FF, V_OC_, and J_SC_ [62,63,64]. Recombination occurs mainly across the interface between the absorber layers (perovskite), and the charge transporting layers which cause the drop of V_OC_ and current. When PEDOT: PSS is used as HTM, the reduction of FF of the device could be attributed to the presence of PSS aggregate in the MAPI: Starch film (Figure 1) and to the degradation of the PEDOT: PSS/MAPI: Starch interface as well; both of these factors increase the recombination rate of photogenerated electrons and holes at the interface between perovskite and PEDOT: PSS. In absence of PSS, concerning PEDOT, albeit the formation of a good interface between HTM and MAPI: Starch, without the presence of aggregates into perovskite film, the corresponding devices feature similar V_OC_ and a much lower photogenerated current that can be attributed in this case to the much lower work function thus unfavourable energy level alignment (see Figure 5). Additionally, the different film growth mechanism, resulting in different morphology of the resulting perovskite, could play a role in affecting the performances. Due to the small size of the grains, the number of boundaries increases, leading to larger carriers-superficial traps deactivation processes worsening the device performances. Voc increases with a more suitable energy alignment, as in the case of PTAA and poly-TPD. However, PTAA based devices produce less current, possibly due to a non-optimized thickness of this layer and/or to a difference perovskite growing mechanism derived from its diverse polarity leading to a reduced carriers extracting interface. The best performances were achieved by the device based on poly-TPD as HTM showing a PCE of 15.1% with a short-circuit current density (J_SC_) of 20.35 mA/cm^–2^, a V_OC_ of 1.06 V, and a fill factor FF of 70%. The correlations between the photovoltaic parameters of MAPI/starch solar cell with different HTLs film are summarized and presented in Figure 6.

Since Poly-TPD showed the best performances, in order to further optimize the PSC performances we focused on this HTM to investigate the effect of polymer characteristics, such as the molecular weight (MW) which is known to influence the solubility, film formation, and carrier mobility [65]. To further explore the influence of wettability, in addition we applied on Poly-TPD a stronger hydrophilic treatment, oxygen plasma.

We compared Poly-TPD already employed in the first part of our study, characterized by a MW < 10 kDa, with a higher MW Poly-TPD (later named HMW) with a MW > 20 kDa.

**Figure 7:**
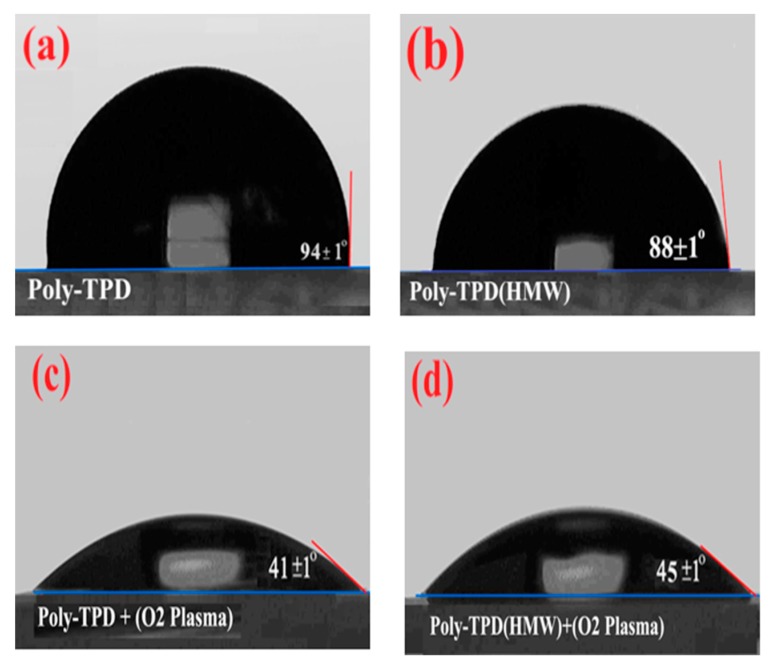
Water contact angle of (**a**) Poly-TPD, (**b**) Treated Poly-TPD High Molecular weight (HMW), (**c**) Treated Poly-TPD with O_2_ plasma, and (**d**) Treated Poly-TPD (HMW) with O_2_ plasma.

In Figure 7 the contact angle of the treated Poly-TPD at different MW before and after 5 s of oxygen plasma treatment are shown. The oxygen plasma seems to have a greater effect on the wettability of the Poly-TPD surface than UVO, in both cases, because the contact angle of the water with Poly-TPD surface decreased to <50°. The decrease in the contact angle of Poly-TPD after treatment indicates an increase in the polarity of the surface of this polymer [66].

**Figure 8:**
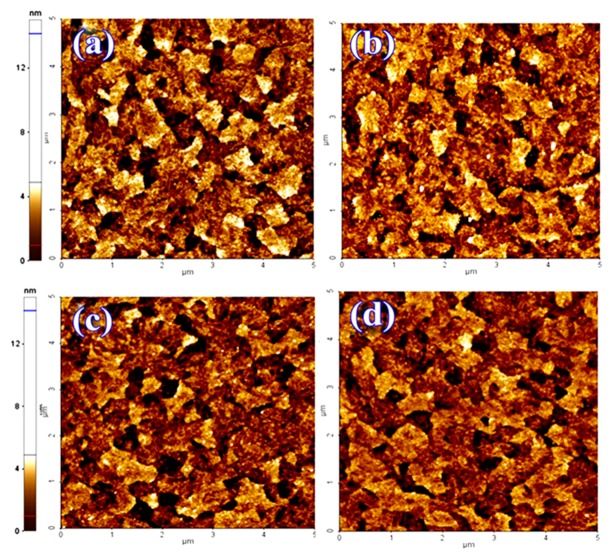
AFM images (5 × 5 μm) of the treated Poly-TPD (Rq = 1.79 nm) (**a**), the treated Poly-TPD (HMW) (Rq = 2.00 nm) (**b**), the treated Poly-TPD with Oxygen plasma (Rq = 1.72 nm) (**c**), and the treated Poly-TPD (HMW) with Oxygen plasma (Rq = 1.86 nm) (**d**).

Figure 8 shows that the surface roughness of perovskite: Starch films deposited on the treated Poly-TPD (LMW and HMW) films with UVO and Oxygen plasma is essentially unchanged.

**Figure 9:**
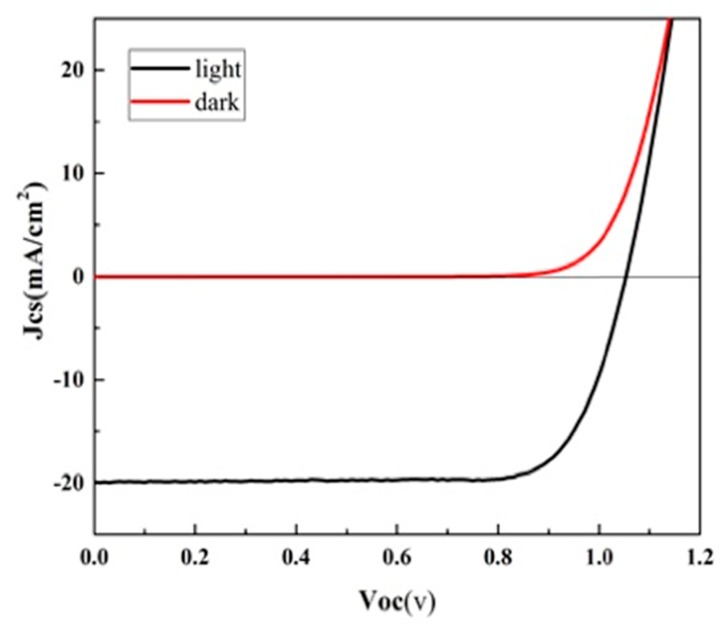
Current–voltage characteristics of champion devices based on perovskite/starch film deposited on the treated Poly-TPD (HMW) layer by UV-Ozone (UVO).

Devices with the architecture Glass/ITO/treated Poly-TPD (HMW and LMW)/Perovskite: Starch/PCBM/BCP/Al, as described in the experimental section, were made. The average measured photovoltaic parameters and the current-voltage curves related to perovskite solar cells constructed by the Poly-TPD at different MWs treated by UVO and Oxygen plasma is shown in Table 2, while the IV curve of the champion device is shown in Figure 9. At best, the photovoltaic parameters of this device are as follows: J_SC_ = 19.9 mA/cm ^2^, V_OC_ = 1.05 V, FF = 78%, and PCE of 16.34%, with respect to PCE = 15.1%, FF = 70%, J_SC_ = 20.35 mA/cm^2^, V_OC_ = 1.06 V of the low molecular weight poly-TPD. The FF is greatly improved, as well as the statistical distribution of the PV parameters around the average value.

The relationship between FF and MW is related to recombination of electrons and holes and to the charge transport across the layer, which appears to improve for higher molecular weight.

Although the hydrophilicity of the film under oxygen plasma was greatly improved, the performance of these cells greatly decreased, indicating a partial decomposition of the transporting polymer.

## 4. Conclusions

In summary, we showed that the engineering of the interface between a perovskite/starch composite and charge transporting layers requires the choice of the appropriate hole transporting layers in inverted planar perovskite solar cells.

We studied inverted planar PSCs realized exploring different polymers; we found that the use of Poly-TPD provides a better interface between the Poly-TPD and perovskite-polymer composite layer, by reducing recombination and improving electron and hole transport, which eventually leads to an increase in the performance of the device (PCE = 15%). The photovoltaic characteristics of the solar cells, especially the FF, show a slight dependence on the Poly-TPD MW, allowing the obtainment of a champion device with high molecular weight Poly-TPD (PCE = 16.3%).

## Figures and Tables

**Table 1:** The average measured photovoltaic parameters for inverted planar perovskite solar cell with different polymeric hole transporting layers (HTLs) under AM1.5G illumination (~100 mW/cm^−2^).

HTL	FF (%)	V_oc_ (V)	J_sc_ (mA/cm^2^)	PCE (%)
**PEDOT: PSS**	60.4 ± 8.4	0.89 ± 0.03	18.0 ± 3.5	9.6 ± 1.7
**PEDOT**	71.1 ± 3.5	0.87 ± 0.01	11.6 ± 0.9	7.2 ± 0.6
**PTAA**	69.2 ± 8.1	1.06 ± 0.03	11.7 ± 2.0	8.5 ± 0.8
**Poly-TPD**	72.4 ± 6.7	1.05 ± 0.03	18.5 ± 2.3	14.0 ± 1.1

**Table 2:** The average measured photovoltaic parameters for inverted planar perovskite solar cell with Poly-TPD (HMW) under AM1.5G illumination (~100 mW cm^2^).

Conditions	FF (%)	V_OC_ (V)	J_SC_ (mA/cm^2^)	PCE (%)
Treated Poly-TPD with UVO	72.4 ± 6.7	1.05 ± 0.03	18.5 ± 2.3	14.0 ± 1.1
Treated Poly-TPD (HMW) with UVO	76.8 ± 2.4	1.05 ± 0.02	18.5 ± 1.0	15 ± 1.5
Treated Poly-TPD with O_2_ Plasma	43.3 ± 3	1.04 ± 0.03	14.3 ± 1.3	6.5 ± 1.0
Treated Poly-TPD (HMW) with O_2_ Plasma	47 ± 1	1.05 ± 0.01	15.6 ± 1.3	7.8 ± 0.9
